# Independent and Combined Effects of Probiotics and Prebiotics as Supplements or Food-Rich Diets on a Propionic-Acid-Induced Rodent Model of Autism Spectrum Disorder

**DOI:** 10.3390/metabo13010050

**Published:** 2022-12-29

**Authors:** Sana Razhan M. Alsubaiei, Hanan A. Alfawaz, Abdullah Yaseen Almubarak, Nouf Ahmed Alabdali, Abir Ben Bacha, Afaf El-Ansary

**Affiliations:** 1 Department of Food Science and Nutrition, College of Food & Agriculture Sciences, King Saud University, P.O. Box 22452, Riyadh 11495, Saudi Arabia; 2 Experimental Surgery and Animal Lab, College of Medicine, King Saud University, P.O. Box 2925, Riyadh 11461, Saudi Arabia; 3 Biochemistry Department, Science College, King Saud University, P.O. Box 22452, Riyadh 11495, Saudi Arabia; 4 Central Research Laboratory, Female Center for Medical Studies and Scientific Section, King Saud University, P.O. Box 22452, Riyadh 11495, Saudi Arabia

**Keywords:** autism spectrum disorder (ASD), propionic acid, yogurt, *Lacticaseibacillus rhamnosus GG*, luteolin, artichoke, probiotics, prebiotics

## Abstract

The link between nutrition and autism spectrum disorder (ASD) as a neurodevelopmental condition, which is clinically presented as significant delays or deviations in interaction and communication, has provided a fresh point of view and signals that nutrition may play a role in the etiology of ASD, as well as playing an effective role in treatment by improving symptoms. In this study, 36 male albino rat pups were used. They were randomly divided into five groups. The control group was fed only a standard diet and water for the 30 days of the experiment. The second group, which served as a propionic acid (PPA)-induced rodent model of ASD, received orally administered PPA (250 mg/kg body weight (BW)) for 3 days, followed by feeding with a standard diet until the end of the experiment. The three other groups were given PPA (250 mg/kg body weight (BW)) for 3 days and then fed a standard diet and orally administered yogurt (3 mL/kg BW/day), artichokes (400 mL/kg BW/day), and a combination of *Lacticaseibacillus rhamnosus* GG at 0.2 mL daily (1 × 10^9^ CFU; as the probiotic of yogurt) and luteolin (50 mg/kg BW/day; as the major antioxidant and anti-inflammatory ingredient of artichokes) for 27 days. Biochemical markers, including gamma-aminobutyric acid (GABA), reduced glutathione (GSH), glutathione peroxidase (GPx1), tumor necrosis factor-alpha (TNF-α), interleukin-6 (IL-6), and interleukin-10 (IL-10), were measured in brain homogenates in all groups. The data showed that while PPA demonstrated oxidative stress and neuroinflammation in the treated rats, yogurt, *Lacticaseibacillus rhamnosus GG* as a probiotic, and luteolin as a prebiotic ingredient in artichokes were effective in alleviating the biochemical features of ASD. In conclusion, nutritional supplementation seems to be a promising intervention strategy for ASD. A combined dietary approach using pro- and prebiotics resulted in significant amelioration of most of the measured variables, suggesting that multiple interventions might be more relevant for the improvement of biochemical autistic features, as well as psychological traits. Prospective controlled trials are needed before recommendations can be made regarding the ideal ASD diet.

## 1. Introduction

Autism spectrum disorder (ASD) is a prevalent neurodevelopmental disorder with substantial clinical heterogeneity. The role of neuroinflammation in ASD has become increasingly evident, and previous studies have demonstrated neuroinflammation in the cerebral cortex, cerebellum, and white matter of patients with ASD. Furthermore, the cerebrospinal fluid (CSF) and serum of living patients with ASD show significantly higher proinflammatory cytokine profiles.

The gut–brain axis, which refers to the bidirectional route between gut bacteria and the brain, has a significant impact on numerous brain processes. These mechanisms include oxidative stress; neuroinflammation; glutamate excitotoxicity; blood–brain barrier construction; neurogenesis; microglia maturation, GABA, noradrenaline, and dopamine synthesis; and behavioral variance, which is a key component of ASD [1,2,3,4].

Because there is no cure for autism, treatments often focus on speech and behavioral interventions to address the disorder’s hallmark social, behavioral, and communication difficulties [5]. Gastrointestinal (GI) disturbances are prevalent comorbidities that are thought to be both a sign of ASD and an etiological cause [6]. The gut microbiota is altered in ASD, with diverse alterations described at different taxonomic levels, highlighting the necessity of examining the gut–brain axis in the treatment of these disorders [7].

In numerous investigations involving human and animal models of autism, dysbiosis of the gut microbiota has been shown to exist. These investigations have revealed that in ASD, aberrant bacterial species prefer the environment of the gut. Biopsy samples from children with ASD have been shown to include abnormal Firmicutes-to-Bacteroidetes ratios [8,9,10]. The imbalance between these two bacterial families varied throughout the various compartments of the gut, which was linked to compositional dysbiosis [11]. Exhibiting higher amounts of proteobacteria [12], lactobacillus [13], bacteroides [14], desulfovibrio [15], and clostridium [16], patients with ASD consistently demonstrate a dysfunctional imbalance. Reduced abundances of bifidobacterium [17], dialister [18], prevotella [19], veillonella, and turicibacter [18] are frequently observed in conjunction with this. Consequently, nutritional interventions are used by the majority of patients with ASD, both with and without clinical management, to relieve GI and behavioral symptoms. Despite considerable interest in dietary interventions, no agreement has been reached regarding optimal nutritional intervention strategies [19].

Food choices and dietary patterns are also suggested to play a role in the development of ASD. Recent evaluations have emphasized the importance of nutrition in regulating or lowering ASD symptoms. It is generally known that consuming prebiotics and probiotics provides various health benefits by positively modifying gut flora. People with autism spectrum disorder (ASD) have an imbalanced gut microbiota. The use of probiotics, prebiotics, and synbiotics is a promising technique for regulating the gut flora and lowering ASD symptoms [20]. Despite the infrequency of studies related to the supplementation of probiotics and prebiotics in individuals with ASD, a promising improvement has been noted in the severity of social interactions associated with an increase in beneficial bacteria and a decrease in pathogens in the GI tract, leading to an improvement in recurrent GI problems, suggesting both pre- and probiotics as promising alternative complementary medicine [19,20].

There is increasing evidence regarding the use of inulin as a prebiotic for the selective growth of bifidobacteria and lactobacilli as beneficial gut bacteria linked to several health benefits. Costabile et al. [21] reported that the daily consumption of inulin extracted from globe artichokes exerts a pronounced prebiotic effect on the composition of human fecal microbiota. Although a pronounced variation in chemical composition and nutritional value was observed in different artichoke genotypes, all have high nutritional value and are significantly recommended as part of a healthy and balanced diet [22].

Luteolin (3′,4′,5,7-tetrahydroxyflavone) is well known as a common component in plants. Luteolin-rich plants have been used ethnopharmacologically for the treatment of inflammation. Both luteolin supplements and extracts from luteolin-rich plants, such as artichokes, have been studied using several models and demonstrated anti-inflammatory activity [23].

Yogurt, fermented milk, and fermented vegetables are all excellent probiotic sources [24]. Consuming probiotics may be advantageous for the improvement of neurological and neurodevelopmental diseases, such as ASD, because the gut microbiota has been found to have a bidirectional link with the brain [25].

The formation of short-chain fatty acids, such as PPA, by intestinal clostridia and desulfovibrio is thought to play a role in the development of ASD symptoms [26]. The PPA model shows several characteristics that are typical of children with autism. In the PPA model, increasing oxidative stress and free radicals cause mitochondrial malfunction, which releases potent cytokines that irritate and change several neurotransmitters. Additionally, the PPA model and patients with ASD are found to share pathophysiological similarities and gastrointestinal problems. Using appropriate behavior testing and modeling criteria, multiple studies have demonstrated that PPA can fulfil more than three aspects. The PPA model offers the most difficult situation and affects a specific brain area to make it the closest one to autism and distinguish it. It is also regarded as a low-cost and simple method of testing novel treatments [26,27,28]. Most recently, Ali et al. [29] proved the validity of the PPA model of ASD.

This information sparked our interest in examining the ability of luteolin and lactobacillus, either as supplements or in food-rich sources, such as artichoke or yogurt, to ameliorate specific biochemical variables related to oxidative stress, neurochemistry, and neuroinflammation, which are the three major etiological mechanisms of ASD, as well as the biomarkers of PPA-induced neurotoxicity in rodent models of ASD [30]. Also investigated was a combined pre- and probiotic intervention (*L. rhamnosus* GG plus luteolin). It is crucial to emphasize that the same nutritional interventions significantly improved the gut microbiomes of ASD animal models that had been induced by PPA (unpublished work under review).

## 2. Results

### 2.1. Effect of the Nutritional Interventions on GPX1 and GSH as Oxidative Stress Markers and the Impaired GABA Neurotransmitter

**Figure 1:**
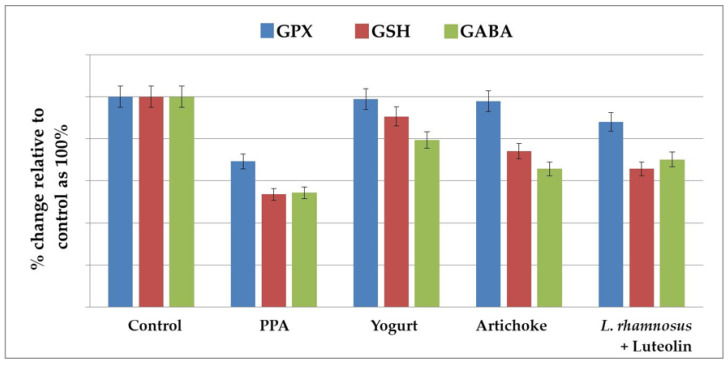
The percentage change in GPX, GSH, and GABA levels in the brain homogenates of an untreated PPA-induced autism model and nutritionally treated groups of yogurt, artichokes, and combined *L. rhamnosus* + luteolin relative to the control, presented as 100%.

**Figure 2:**
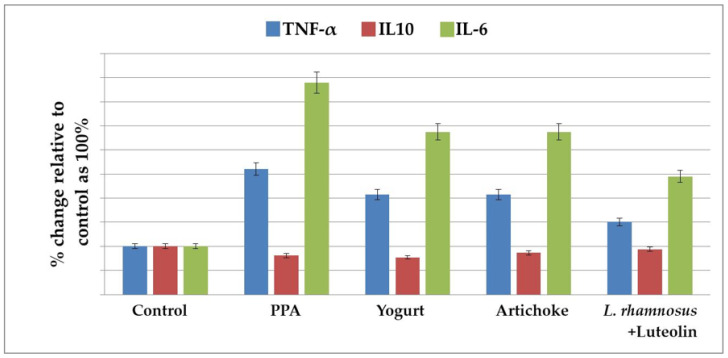
The percentage change in TNF-α, IL10, and IL-6 levels in the brain homogenates of an untreated PPA-induced autism model and nutritionally treated groups of yogurt, artichokes, and combined *L. rhamnosus* GG + luteolin relative to the control, presented as 100%.

Data are presented as means ± S.D., together with the percentage changes in all the measured variables (Table 1 and Table 2 and Figure 1 and Figure 2). Table 1 and Figure 1 demonstrate a significant decrease in GPX1 (−32.78%), GSH (−52.17%), and GABA (−28.83%) in the PPA-treated group as a rodent model of ASD, together with the remarkable ameliorative effects of the four nutritional interventions used in treatments. Although artichokes remarkably increased GPX1 and GSH in PPA-treated rats, the PPA-treated rats still demonstrated significantly lower GSH levels compared to controls (−25.95%). In contrast, the yogurt-treated group recorded more or less similar GSH and GPX1 levels to those of the controls and significantly higher levels compared to the PPA-treated group.

### 2.2. Effect of the Nutritional Interventions on the Levels of TNF-α, IL-10, and IL-6 as Neuroinflammatory Markers

Table 2 and Figure 2 demonstrate the levels of TNF-α, IL-10, and IL-6 in the five studied groups. PPA treatment induced a highly significant increase in TNF-α and IL-6 as pro-inflammatory cytokines, recording percentage increases of 196.81% and 482.8%, respectively, compared to the control group of rats. IL-10, as an anti-inflammatory cytokine, was significantly lower in the PPA-treated group and did not increase after nutritional interventions. Yogurt, artichoke, and *L. rhamnosus* GG + luteolin treatments demonstrated significantly lower levels of TNF-α and IL-6 compared to the PPA-treated group, but these levels were still remarkably higher than in the controls. The *L. rhamnosus* GG + luteolin-treated group recorded the lowest values of both pro-inflammatory cytokines.

### 2.3. Receiver Operating Characteristic Analysis for Evaluating Predictive Values of the Measured Variables in the PPA-Induced Autism Model and Different Nutritionally Treated Groups

Table 3 and Table 4 demonstrate the area under the curves (AUCs) of the receiver operating characteristics (ROC) curves, cut-off values, specificity, and sensitivity of the six measured variables in all the groups. It can be noted that most of the parameters recorded high AUCs, together with satisfactory specificity and sensitivity.

## 3. Discussion

It is well accepted that the etiology of ASD may involve complex interactions between genetic factors and certain environmental toxicants that may act synergistically or in parallel during critical periods of neurodevelopment, increasing the likelihood of developing ASD in at least a subset of children.

The current treatment of psychiatric disorders primarily focuses on the use of psychotropic medicine to treat symptoms, although its efficiency varies between people, and it is typically linked with severe adverse effects. In recent years, nutritional therapies for the prevention and treatment of mental diseases have gained a lot of attention. However, data supporting nutritional interventions in autism spectrum disorder are still limited and of poor quality [31].

The lower recorded GPX1 activity in the combined lactobacillus-and-luteolin-treated group compared to the independently treated yogurt and artichoke groups could be attributed to the fact that luteolin, as a prebiotic, quenches ROS and prevents their damaging effects on brain cells and that lactobacilli, as excellent organic acid producers, convert sugars into lactic acid and other by-products, including H_2_O_2_, a substrate of GPX1, which could affect its enzymatic activity [32]. They produce small molecules as well. Regarding GABA, there was a significant decrease in PPA-treated groups, together with a remarkable elevation in the yogurt-treated group, whereas both the artichoke and L. rhamnosus GG + luteolin treatments were ineffective in inducing GABA levels.

Many children with ASD have been observed to suffer from co-morbidities, such as GI distress and abnormal sensory processing, which may restrict their nourishment. To compensate for nutritional deficiencies attributable to the reduction in food selectivity and the abnormal eating habits of patients with ASD, several dietary strategies have been applied by caregivers, such as the supplementation of diets with probiotics, a large amount of fiber, omega-3 fatty acids, antioxidants, and vitamins and minerals, but most of these are still confusing and inconclusive [33].

There is growing interest in the use of combined prebiotics, such as oligosaccharides, and probiotics to support human health. Combining these two to create a successful synbiotic could maximize their therapeutic effects. Simply, prebiotics can improve the composition of the gut microbiome, support the immune system by increasing the number of protective microorganisms, and reduce the number of harmful or pathogenic microorganisms [19,20]. In this study, we investigated selected nutritional intervention strategies using a PPA-induced animal model of ASD [28,30]. Among these strategies are pure probiotics, probiotic-rich food, fiber- and flavonoid-rich food, and luteolin either independently or in combination.

Previous research has found that children with ASD have reduced GSH levels. Nutritional therapies aimed at increasing GSH levels have been demonstrated to improve ASD behaviors [34,35]. GSH and GPX1 play a role in the antioxidant defense against a wide range of environmental pollutants, including PPA [36,37,38]. Table 1 and Figure 1 demonstrate the significant decrease in GPX1 and GSH observed in both PPA-treated groups in the rodent model of ASD. This result can find support in the recent work carried out by Al Suhaibani et al. [39], in which PPA-treated animals demonstrated a significant reduction in GSH compared with controls. Additional support can be also found in the previous studies by Macfabe et al. and El-Ansary et al. [30,40], who both reported reductions in GSH in PPA-treated rats. They hypothesized that increased levels of PPA could induce oxidative stress in the brain, first when orally administrated and second when intraventricularly administrated, along with repetitive, social, and object-directed behaviors [40]. Table 1 also presents the antioxidant effects of yogurt, as shown by the amelioration of GSH and GPX depletion. This can find support in the work of Gjorgievski et al. [41], which proved that yogurt fermented with different microbiological cultures, including symbiotic *Lactobacillus* spp., shows health-promoting effects and strong antioxidant activity compared with unfermented milk.

Table 1 and Figure 1 also demonstrate the antioxidant effects of artichokes and luteolin as active ingredients of artichokes. Both demonstrated significant potency in amending the oxidative stress induced by PPA as a neurotoxicant. Luteolin demonstrated higher antioxidant effects compared to whole-artichoke extracts. This finding is in good agreement with the previous work in [42], which proved that artichoke leaf extract displays high potential as a natural source of minerals and phytochemical compounds with antioxidant and anti-inflammatory properties. The authors proved that methanol extract from artichokes shows a significant decrease in lipid peroxides as an indicator of oxidative behavior in children given luteolin at 100 mg/capsule per 10 kg (22 lb.) weight per day with food for 26 weeks. They attributed the improvement in behavior to the antioxidant, anti-inflammatory, and neuroprotective effects of luteolin. The remarkable increase in GABA observed in prebiotic- and probiotic-treated groups can find support in multiple previous works that have proven that food-derived *Lactobacillus* strains, such as *Lactobacillus plantarum* [43], *Lactobacillus paracasei*, *Lacticaseibacillus rhamnosus GG* [44], and *Lactobacillus brevis*, are effective in alleviating the decreased GABA levels usually associated with depression and anxiety brain disorders [44].

Chronic neuroinflammation has been identified in ASD [45]. This includes chronic glia activation and changed inflammatory function, which could be somewhat responsible for the abnormal behavior observed in ASD. It is well accepted that chronic peripheral inflammation and abnormal inflammatory responses in the brain may lead to cognitive dysfunction [46]. Table 2 and Figure 2 demonstrate significant increases in the proinflammatory cytokines TN-α and IL-6, together with a non-significant decrease in IL-10 as an anti-inflammatory cytokine, in PPA-treated rats. This result was supported by the most recent work of Abdelli et al. [47] and Abuaish et al. [28], who demonstrated a remarkable increase in gliosis and neuron-inflammatory biomarkers in a PPA-rodent model of ASD. Table 2 and Figure 2 also show the significant therapeutic effects of yogurt, artichokes, luteolin, *L rhamnosus*, and combined *L rhamnosus* + luteolin. This finding is supported by multiple previous studies that have proved that probiotic yogurt intake is associated with significant anti-inflammatory effects that parallel the increase in the peripheral pool of T (reg) cells in patients with IBD [48]. Furthermore, yoghurt and its associated probiotics may improve intestinal barrier function by maintaining tight-junction protein expression and aiding in the prevention of gut inflammation and tissue injury [49]. Yogurt containing *Lactobacillus bulgaricus* strains and *Streptococcus thermophilus* strains reduced mortality and prevented chemically induced intestinal inflammation in mice [50]. Furthermore, yoghurt without additional probiotic strains inhibited induced colitis in mice by increasing IgA-producing cells and decreasing CD8+ T-cells 2 weeks after chemical toxin treatment [51]. Based on the fact that leaky gut and tight-junction protein impairment are well-documented features in patients with ASD, this study could help suggest yogurt consumption as a therapeutic strategy working through the gut–brain axis in patients with ASD [52]. The significant therapeutic effects of artichokes shown in Table 2 and Figure 2 are in good agreement with multiple studies that have demonstrated remarkable decreases in pro-inframammary markers in artichoke-treated mice with colitis induced by dextran sulfate sodium [53]. Additionally, Wauquier et al. [54] demonstrated that plant-derived nutrients and especially polyphenols from artichokes may represent a relevant alternative for nutritional strategies addressing multiple inflammatory chronic diseases. The anti-inflammatory effects of luteolin can be easily observed in Table 2 and Figure 2 as significant decreases in TNF-α and IL-6 induced in PPA-treated groups. This is in good agreement with a previous study by Aziz et al. [23], which proved that luteolin, as a flavonoid commonly found in medicinal plants, such as artichokes, has strong anti-inflammatory activity in vitro and in vivo. The anti-inflammatory effects of luteolin occur mostly through the inhibition of the nuclear factor (NF)-κB pathway, mitogen-activated protein kinase (MAPK), and signal transducer and activator of transcription 3 (STAT3). Additionally, a clinical trial with a formulation containing luteolin revealed therapeutic effects against multiple inflammation-associated diseases. Luteolin, as a component of artichokes, demonstrates remarkably higher anti-inflammatory effects than whole-plant extract. Probiotic microorganisms are thought to benefit human health primarily through three basic modes of action [23,55]. First, some probiotics have the ability to remove or suppress pathogens, either directly or through their effects on commensal microbiota [56,57]. A second mechanism is the capacity of specific probiotics to increase epithelial barrier function by regulating signaling pathways, such as NF-kB, Akt, and MAPK, which results in the induction of mucus [58] or improved tight-junction function. Third, most probiotics have the ability to regulate host immunological responses [59]. Many interactions between probiotic bacteria and intestinal epithelial and immune cells are hypothesized to be mediated by molecular structures known as microbe-associated molecular patterns (MAMPs), which can be identified by pattern recognition receptors (PRRs), such as TLRs [60]. *L rhamnosus* is one of the most commonly used probiotics, demonstrating both antioxidant and anti-inflammatory effects in this study (Table 1 and Table 2 and Figure 1 and Figure 2). These findings are supported by the work of Ayyanna et al. [61], who observed *L. rhamnosus GG*-induced downregulation of pro-inflammatory cytokines, including IL-6, and significant decreases in lipid peroxides and ROS as markers of oxidative stress [62].

The therapeutic effects of pre- and probiotics observed in this study can find support in multiple recent studies that have demonstrated the effectiveness of prebiotics and prebiotics used as psychobiotics in treating the symptoms of schizophrenia and its comorbidities, attention deficit hyperactivity disorder (ADHD), bipolar disorder, and other neuropsychiatric disorders affecting children and adolescents [63,64,65,66]. Although still in its early stages, the use of prebiotics and probiotics to treat the symptoms of neurological disorders is quite promising.

Table 3 demonstrates that ROC curve analysis is an appropriate statistical tool for evaluating both the sensitivity and specificity of a biochemical variable or biomarker. It helps ascertain optimal cut-off points for a measured variable for potential follow-ups for future clinical applications [67]. The absence of false positives and false negatives for any measured variable means that this method demonstrates perfect performance. ROC analysis produces an AUC, which is a measure of how well a parameter can discriminate between two studied groups (i.e., PPA-intoxicated or prebiotic- and probiotic-treated groups relative to controls in this study). The AUC usually ranges from 0.5 (no discriminant capacity) to 1.0 (perfect discriminant capacity) [67].

## 4. Materials and Methods

### 4.1. Materials

In March 2019, fresh *Cynara scolymus* L. (artichokes) exported from the Netherlands was purchased from local supermarkets in Riyadh, Saudi Arabia (SA). Yogurt was purchased from local supermarkets in Riyadh, Saudi Arabia. Following collection, the samples were stored aseptically in a refrigerator at a low temperature (4 °C) to preserve them from contamination and deterioration [53]. Probiotic *Lacticaseibacillus rhamnosus GG* and prebiotic luteolin supplements were purchased from Swanson Health Products (Fargo, ND, USA).

### 4.2. Preparation of Cynara Scolymus L. (Artichoke) Extract

*Cynara scolymus* L. heads were divided into petal, choke, and heart sections. Each component was cleaned, cut, shade-dried at room temperature, and then processed into powder in a coffee grinder. The petal, choke, and heart total dry powder weights were 3.16 kg, 1.21 kg, and 0.371 kg, respectively. The powder of the *Cynara scolymus* L. (artichoke) head petal, choke, and heart was extracted separately with methanol/water (80/20, V/V) over 72 h using an orbital shaker at 150 rpm. Next, it was filtered through Whatman paper and re-extracted four times using a new solvent (methanol/water). The artichoke extract in the flask was immersed in a water bath during the evaporation process. The extract was placed in the hood for 24 h to ensure complete methanol evaporation, and then, a few drops of chloroform were added to prevent fungal contamination. The final dry extract was stored at 4 °C until further use. 

### 4.3. Animals

Thirty-six male Sprague–Dawley albino rat pups were used, with an average weight of 70 g ± 20 g (approximately 3 weeks old). They were divided into 5 groups randomly. The control group was fed only a standard diet and water for the 30 days of the experiment. The second group served as a PPA-induced rodent model of ASD, orally administered PPA (250 mg/kg body weight (BW)) for 3 days, followed by feeding with a standard diet until the end of the experiment. The three other groups were given PPA(250 mg/kg body weight (BW)) for 3 days and then fed a standard diet and orally administered yogurt (3 mL/kg BW/day) [68], artichokes (400 mL/kg BW/day) [69], and a combination of *L. rhamnosus* GG 0.2 mL daily (1 × 10^9^ CFU) [56] and luteolin (50 mg/kg BW/day) [70,71,72] for 27 days. The biochemical markers glutamate, gamma-aminobutyric acid (GABA), glutathione (GSH), glutathione peroxidase 1(GPX1), tumor necrosis factor-alpha (TNF-α), interleukin 6 (IL6), and interleukin 10 (IL10) were measured in brain homogenates in all the groups. The Graduate Studies and Scientific Research Ethical Committee of Bioethics of King Saud University (KSU; reference no. SE-19-142) accepted the protocol for this study, and it was carried out in accordance with its rules. Our investigation was conducted in accordance with the ARRIVE recommendations. The 5 study groups are shown in Table 5 including the control group, the PPA-induced rodent model, and the three nutritionally treated groups.

### 4.4. Preparation of Brain Tissue Homogenates

Deeply anaesthetized (with ketamine/xylazine + D.W. (91, respectively 9 mg/kg BW, I.P.) animals were beheaded at the end of the feeding sessions. Brain tissues were extracted from the five groups of rats and dissected into minute pieces before being homogenized in bi-distilled water (1:10, *w*/*v*) and kept at −30 °C until further use.

### 4.5. Biochemical Analyses

#### 4.5.1. Determination of GSH

GSH was measured in brain homogenates using a competitive ELISA kit (GPX1; Cat.No: CEA294Ge; Cloud Clone Corp., 23603 W. Fernhurst Dr., Unit 2201, Katy, TX 77494, USA). The assay was performed according to the manufacturer’s protocols. Its sensitivity is typically less than 0.52 μg/mL.

#### 4.5.2. Determination of GPX1

GPX1 was measured in brain homogenates using a competitive ELISA kit (GPX1; Cat.No: SEA295Ra; Cloud Clone Corp., 23603 W. Fernhurst Dr., Unit 2201, Katy, TX 77494, USA). The assay was performed according to the manufacturer’s protocols. Its sensitivity is typically less than 0.61 ng/mL.

#### 4.5.3. Determination of GABA

GABA was measured in brain homogenates using a competitive ELISA kit (GPX1; Cat.No: CEA900Ge; Cloud Clone Corp., 23603 W. Fernhurst Dr., Unit 2201, Katy, TX 77494, USA). The assay was performed according to the manufacturer’s protocols. Its sensitivity is typically less than 2.17 pg/mL. Samples were measured at a wavelength of 450 nm ± 10 nm.

#### 4.5.4. Determination of IL-6

A competitive ELISA kit (GPX1; Cat. No. SEA079Ra; Cloud Clone Corp., 23603 W. Fernhurst Dr., Unit 2201, Katy, TX 77494, USA) was used to quantify IL-6 in brain homogenates. The test was conducted in accordance with the manufacturer’s instructions. Typically, its sensitivity is lower than 3.3 pg/mL.

#### 4.5.5. Determination of IL-10

IL-10 was measured in brain homogenates using a competitive ELISA kit (GPX1; Cat.No: SEA056Ra; Cloud Clone Corp., 23603 W. Fernhurst Dr.; Unit 2201; Katy; TX 77494; USA). The assay was performed according to the manufacturer’s protocols. Its sensitivity is typically less than 5.8 pg/mL.

### 4.6. Statistical Analyses

The data are presented as means ± standard deviations. All statistical comparisons between the control group and the PPA- and probiotic-treated rat groups were made using SPSS Statistics version 16.0, with one-way analysis of variance (ANOVA) tests, together with Dunnett’s test for multiple comparisons. The threshold for significance was set at *p* < 0.05. Analysis of the ROC curve was also carried out. Calculations were carried out to determine the AUCs, levels of sensitivity and specificity, and cut-off values.

## 5. Conclusions

Taken together, these results support the potential effectiveness of probiotic (*Lacticaseibacillus rhamnosus GG*) and prebiotic (artichoke and Luteolin) treatments, either independently or in combination, as nutritional intervention strategies to amend oxidative stress and neuroinflammation as neurotoxic effects of an orally administered PPA rodent model of ASD.

## 6. Future Perspectives

In the light of the effectiveness of probiotics and prebiotics that have been used as supplements or food-rich diets to improve induced biochemical autistic features, we expect that future studies will be able to assess the whole physiological effects of these diets. Such information can direct the development of interventions that are more informed, less constrictive, free from the negative effects of limiting certain nutrients, and still keep the components that promote the beneficial behavioral amendment of ASD.

## Figures and Tables

**Table 1:** Effect of nutrition with yogurt, artichokes, and combined *L. rhamnosus* GG + luteolin on levels of GPX1 (U/mg protein), GSH (µg/mg protein), and GABA (Pg/mg protein) in the brain homogenates of the PPA-induced rodent model of autism.

Parameters	Groups	Mean ± S.D.	*p*- Value
**GPX**	**Control**	108.60 ± 11.05	0.001
	**PPA**	75.19 ± 9.72 a	
	**Yogurt**	107.33 ± 15.44 b	
	**Artichoke**	106.21 ± 18.80 b	
	***L. rhamnosus* GG + Luteolin**	95.48 ± 14.47 b	
**GSH**	**Control**	15.18 ± 2.27	0.001
	**PPA**	8.13 ± 1.82 a	
	**Yogurt**	13.75 ± 2.97 b	
	**Artichoke**	11.24 ± 2.08 ab	
	***L. rhamnosus* GG + Luteolin**	9.97 ± 1.75 ab	
**GABA**	**Control**	65.91 ± 10.41	0.004
	**PPA**	35.80 ± 5.82 a	
	**Yogurt**	52.33 ± 18.13 b	
	**Artichoke**	43.29 ± 12.07 a	
	***L. rhamnosus* GG + Luteolin**	46.22 ± 17.77 a	

(a) A significant difference between the group and the control group at a significance level of *p* = 0.05; (b) a significant difference between the group and the PPA group at a significance level of *p* = 0.05.

**Table 2:** Effect of nutrition with yogurt, artichokes, and combined *L. rhamnosus* GG + luteolin on levels of TNF-α (pg/mg protein), IL10 (pg/mg protein), and IL-6 (pg/mg protein) in the brain homogenates of the PPA-induced rodent model of autism.

Parameters	Groups	Mean ± S.D.	*p*-Value
**TNF-α**	**Control**	14.40 ± 3.53	0.001
	**PPA**	37.53 ± 8.89 a	
	**Yogurt**	29.85 ± 6.84 ab	
	**Artichoke**	29.86 ± 7.93 ab	
	***L. rhamnosus* GG + Luteolin**	21.69 ± 4.67 ab	
**IL10**	**Control**	49.74 ± 3.50	0.331
	**PPA**	40.11 ± 6.76	
	**Yogurt**	38.41 ± 2.50 a	
	**Artichoke**	43.07 ± 7.64	
	***L. rhamnosus* GG + Luteolin**	46.79 ± 5.03	
**IL-6**	**Control**	10.86 ± 0.80	0.001
	**PPA**	47.69 ± 9.33 a	
	**Yogurt**	36.60 ± 8.39 ab	
	**Artichoke**	36.61 ± 9.73 ab	
	***L. rhamnosus* GG + Luteolin**	26.60 ± 5.73 ab	

(a) A significant difference between the group and the control group at a significance level of *p* = 0.05; (b) a significant difference between the group and the PPA group at a significance level of *p* = 0.05.

**Table 3:** ROC results of GPX, GSH, and GABA in the tissue homogenates of the PPA-induced rodent model of autism and yogurt, artichoke, and combined *L. rhamnosus* GG + luteolin nutritionally treated groups relative to the control group.

	Groups	AUC	Cut-Off Value	Sensitivity %	Specificity %	*p*- Value
**GPX1**	**PPA**	1.000	92.785	100.0%	100.0%	0.004
	**Yogurt**	0.833	95.660	66.7%	100.0%	0.055
	**Artichoke**	0.667	99.800	50.0%	83.3%	0.337
	*L. rhamnosus* GG **+ Luteolin**	0.667	95.780	50.0%	100.0%	0.337
**GSH**	**PPA**	1.000	11.905	100.0%	100.0%	0.004
	**Yogurt**	0.889	12.095	66.7%	100.0%	0.025
	**Artichoke**	1.000	12.860	100.0%	100.0%	0.004
	*L. rhamnosus* GG **+ Luteolin**	1.000	11.865	100.0%	100.0%	0.004
**GABA**	**PPA**	0.889	60.800	100.0%	83.3%	0.025
	**Yogurt**	0.833	60.005	83.3%	83.3%	0.055
	**Artichoke**	0.917	62.190	100.0%	83.3%	0.016
	*L. rhamnosus* GG **+ Luteolin**	0.972	62.190	100.0%	83.3%	0.006

**Table 4:** ROC results of TNF-α, IL-10, and IL-6 in the tissue homogenates of the PPA-induced rodent model of autism and yogurt, artichoke, and combined *L. rhamnosus* GG + luteolin nutritionally treated the groups relative to the control group.

	Groups	AUC	Cut-Off Value	Sensitivity %	Specificity %	*p*- Value
**TNF-α**	**PPA**	1.000	26.105	100.0%	100.0%	0.004
	**Yogurt**	0.972	17.235	100.0%	83.3%	0.006
	**Artichoke**	0.861	18.955	66.7%	100.0%	0.037
	***L. rhamnosus* GG + Luteolin**	0.861	19.090	83.3%	100.0%	0.037
**IL-10**	**PPA**	1.000	87.335	100.0%	100.0%	0.004
	**Yogurt**	0.944	72.205	83.3%	100.0%	0.010
	**Artichoke**	0.917	57.470	100.0%	66.7%	0.016
	***L. rhamnosus* GG + Luteolin**	0.722	58.015	83.3%	66.7%	0.200
**IL-6**	**PPA**	1.000	26.610	100.0%	100.0%	0.004
	**Yogurt**	1.000	17.240	100.0%	100.0%	0.004
	**Artichoke**	1.000	14.960	100.0%	100.0%	0.004
	***L. rhamnosus*\GG + Luteolin**	1.000	14.125	100.0%	100.0%	0.004

**Table 5:** Number of animals and the pro/prebiotic dosages fed to animals post orally administered PPA (250 mg /kg BW for 3 day).

Group	No. of Rats	Pro-/Prebiotic Nutritional Interventions	Dosage
1 ^a^	6	-	-
2 ^b^	6	-	(250 mg PPA/kg BW) for 3 days
3	6	Yogurt ^c^	(3 mL/kg BW)
4	6	Artichoke ^d^	(400 mL/kg BW)
5	6	Luteolin and *L. rhamnosus* GG ^e^	(50 mg/kg BW) and 0.2 mL (1 × 10^9^ CFU)

^a^ Control group. ^b^ PPA control group. ^c, d, e^ Received PPA (250 mg/kg BW) for 3 days and were then given nutritional interventions.

## Data Availability

The datasets and analyses generated during this study are available from the corresponding author upon reasonable request. The data are not publicly available due to privacy.
